# Data-driven study on resting-state functional magnetic resonance imaging during early abstinence of alcohol dependence in male patients and its predictive value for relapse

**DOI:** 10.1186/s12888-022-03782-w

**Published:** 2022-02-22

**Authors:** Renhao Deng, Xia Yang, Ya-jing Meng, Yu-jie Tao, Hui-yao Wang, Xiao-jing Li, Wei Wei, Hua Yu, Qiang Wang, Wei Deng, Lian-sheng Zhao, Xiao-hong Ma, Ming-li Li, Jia-jun Xu, Jing Li, Yan-song Liu, Zhen Tang, Xiang-dong Du, Jeremy W. Coid, Andrew J. Greenshaw, Tao Li, Wan-jun Guo

**Affiliations:** 1 Mental Health Center and Psychiatric Laboratory, West China Hospital, Sichuan University, No. 28 Dianxin South Street, Chengdu, 610041 Sichuan China; 2 Department of Clinical Psychology, Suzhou Psychiatric Hospital, The Affiliated Guangji Hospital of Soochow University, Suzhou, Jiangsu China; 3 Department of Psychiatry, University of Alberta, Edmonton, Canada; 4 Center for Educational and Health Psychology, Sichuan University, Chengdu, People’s Republic of China

**Keywords:** Alcohol dependence, Relapse, Magnetic resonance imaging, Predictor

## Abstract

**Background:**

Alcohol dependence is a mental disorder with a high relapse rate. However, specific neuroimaging biomarkers have not been determined for alcohol dependence and its relapse. We conducted data-driven research to investigate resting-state functional magnetic resonance imaging (rs-fMRI) during early abstinence from alcohol dependence and its potential ability to predict relapse.

**Methods:**

Participants included 68 alcohol-dependent patients and 68 healthy controls (HCs). The regional homogeneity (ReHo) and fractional amplitude of low-frequency fluctuations (fALFF) were compared between the alcohol dependence group and the HCs and between the relapse group and the nonrelapse group. The brain regions that presented significantly different ReHo and/or fALFF between the alcohol-dependent patients and HCs and/or between the relapsed and nonrelapsed patients were selected as the seeds to calculate the functional connectivities (FCs).

**Results:**

During a 6-month follow-up period, 52.24% of alcohol-dependent patients relapsed. A regression model for differentiating alcohol-dependent patients and HCs showed that reductions in ReHo in the left postcentral region, fALFF in the right fusiform region, and FC in the right fusiform region to the right middle cingulum were independently associated with alcohol dependence, with an area under the receiver operating characteristic curve (AUC) of 0.841. The baseline FC of the left precentral to the left cerebellum of the relapse group was significantly lower than that of the nonrelapse group. The AUC of this FC to predict relapse was 0.774.

**Conclusions:**

Our findings contribute to advancing research on the neurobiological etiology and predictive biomarkers for relapse associated with alcohol dependence.

## Introduction

According to the global status report from the World Health Organization (WHO), alcohol consumption is the seventh risk factor for death and causes a 5.1% loss of disability-adjusted life years (DALYs) [1]. Approximately 240 million adults worldwide are estimated to suffer from alcohol use disorder (AUD), including alcohol dependence [2]. Alcohol dependence is the inability to control alcohol use and represents a condition in which a person has a craving for or a physical need to consume alcohol, despite its negative impact on their health and family. Alcohol dependence not only is a mental disorder but also contributes to physical diseases. With a high relapse rate, alcohol dependence has become a main reason for the large disease burden worldwide caused by alcohol use [3]. A systematic review indicated that the current prevalence of alcohol dependence in mainland China for males and females was 4.4% and below 0.2%, respectively [4]. Most studies have shown that the relapse of alcohol dependence was higher than 60% [5, 6]. Hence, there is an urgent need to explore the pathogenesis and predictive factors for the relapse of alcohol dependence. Most studies that have investigated the diagnosis and prognostic prediction of alcohol dependence have focused on mainly clinical features, such as clinical symptoms, drinking history and treatment compliance [7–9]. These studies have limited usefulness for elucidating the neurobiological mechanism of alcohol dependence. Moreover, they have reported inconsistent evidence in the prediction of alcohol dependence relapse.

The magnetic resonance imaging (MRI) technique, which is a safe, high-resolution, and noninvasive method, has been increasingly used for the neuroimaging of the mechanisms underlying mental disorders, including addiction [10, 11]. Using MRI techniques, researchers have not only found structural changes, such as abnormal changes in the gray matter [12] and white matter of alcohol-dependent [13] patients but also, observed functional effects. Functional connectivity (FC), which is a fMRI method for observing the functional association between different brain regions by analyzing the statistical correlation between the time series of different brain regions [14], has also been used in alcohol dependence research. For instance, Han et al. (2015) set the dorsolateral prefrontal cortex (DLPFC) as the seed and found that alcohol dependence had positive functional connectivity between the seed DLPFC and cingulate cerebellum [15]. Our previous study, which set the nucleus accumbens (NAc) and medial prefrontal cortex (mPFC) as regions of interest (ROIs), showed that decreased FCs between the NAc and left fusiform cortex as well as between the mPFC and left cingulum anterior cortex might be associated with alcohol dependence [16]. Most of these fMRI studies, especially the functional connectivity studies, were based on the priori hypothesis that defined a brain region that was a result of a previous study as a ROI or seed to investigate alcohol dependence-associated FCs with other brain regions; however, the ROI strategy may ignore brain regions other than ROIs and thus lead to bias [17]. Regional homogeneity (ReHo) and the fractional amplitude of low-frequency fluctuations (fALFF) can be used to conduct whole-brain voxel-wise functional MRI (fMRI) analysis. ReHo is a method of analyzing the local homogeneity of spontaneous blood oxygenation level-dependent (BOLD) signals of fMRI, and it reflects the synchronization in the local brain region by calculating the similarity of the BOLD signals between a voxel and neighboring voxels based on Kendall’s coefficient concordance [18]. Tu et al. (2018) reported that specific brain regions were affected by alcohol, such as the cerebellum, medial frontal gyrus, left precentral gyrus, middle temporal gyrus, inferior temporal gyrus and superior frontal gyrus, which could be indicated by ReHo [19]. fALFF is an index of the rs-fMRI signal that reflects the intensity of spontaneous brain regional activity by calculating the ratio of low-frequency BOLD signals to that of the entire frequency BOLD signals and can be used to explore the functional integrity of the whole brain [20]. For example, Yan et al. (2012) investigated entire-brain fALFF data between alcohol-dependent individuals and controls group and found that the fALFF of the alcohol dependence group was reduced in the bilateral medial prefrontal gyrus and left posterior lobe of the cerebellum and increased in the anterior cingulate and bilateral insular lobes [21]. Although both ReHo and fALFF could reflect whole-brain functional changes in alcohol dependence and a large number of cross-sectional neuroimaging studies have reported functional changes in some brain regions between alcohol-dependent patients and healthy controls (HCs), few longitudinal studies have been performed. Recently, a few studies have investigated brain functional changes related to the relapse of alcohol dependence. Beck et al. (2012) found that in the alcohol-cue stimuli task, the FC of the relapse group between the left medial prefrontal cortex and midbrain was decreased compared with that of the group of abstainers [22]. However, studies on the relapse of alcohol dependence have focus on mainly task-status fMRI. Some researchers have pointed out that the craving occurred in the resting state as well as in the tasking state and have thus indicated that the predictors of relapse of alcohol dependence by resting-state functional magnetic resonance imaging (rs-fMRI) require further investigation [23–27].

To overcome the aforementioned limitations of previous ROI and/or task-status fMRI studies, we conducted data-driven rs-fMRI research to investigate ReHo and fALFF and FCs during alcohol early abstinence and their association with alcohol dependence and relapse over a six-month follow-up period.

## Material and methods

This longitudinal cohort study was approved by the ethics committee of West China Hospital of Sichuan University (2016NO.22). All experiments were performed in accordance with relevant guidelines and regulations. All participants provided a written informed consent.

### Participants

We recruited 136 males of Han nationality to participate in this study: 68 were alcohol-dependent patients and 68 were HCs. Only male participants were included because the prevalence of alcohol dependence is much higher in men than in women [4]. Patients with alcohol dependence were assessed by the Structured Clinical Interview for the Diagnostic and Statistical Manual of Mental Disorders, 4th Edition (DSM-IV) Axis I Disorders-Patient Edition (SCID-I/P) and diagnosed according to the DSM-IV criteria [28]. During screening by trained psychiatric clinicians, participants provided information about the quantity, frequency and drinking pattern of alcohol consumption in their past and current life. The HCs assessed by the diagnostic interview of DSM-IV Axis I Disorders-Nonpatient Edition (SCID-NP) were matched with alcohol-dependent patients in terms of age and education. All participants were right-handed, and those whose intelligence quotient was < 70 or whose age was not in the range of 18 to 55 were excluded. The exclusion criteria for alcohol-dependent patients were a history of or current neurological illness including delirium, comorbidities of other serious mental disorders such as schizophrenia or bipolar disorder, or trauma or substance use disorders except for alcohol or tobacco dependence. The HCs were free of psychotic disorders, emotional disorders or substance-use disorders except tobacco dependence and did not have immediate relatives with psychotic disorders. Patients with alcohol dependence had experienced 5 to 12 days of hospitalized detoxification after stopping drinking alcohol, had been relieved of withdrawal syndromes and could comply with the MRI scanning according to clinicians’ assessment.

### Baseline measurements

Demographic information, including age, education years and smoking status, was collected by a series of self-conducted questionnaires. Clinical characteristics, including age at first drink and number of drinking years, were also measured in the baseline investigation. Alcohol dependence was measured by a Chinese version of the Alcohol Use Disorder Identification Test (AUDIT), which is a short screening tool that made up of 10 questions with total scores ranging from 0 to 40 [29]. Different language versions (including the Chinese version) of AUDIT have been validated [30].

### MRI acquisition

A 3.0 T scanner (Achieva; Philips, Amsterdam, the Netherlands) equipped with an Invivo HD 8-channel high-resolution head coil was used to capture MRI images during early abstinence of alcohol. To minimize scanner noise and head movement, we used earplugs and foam padding. Before scanning, participants were told not to fall asleep but to rest quietly with their eyes closed and to empty their minds (checked with participants while finishing the MRI scans).

BOLD signals were obtained with an echo-planar imaging (EPI) sequence at rest for approximately 8 min. Thirty-eight axial slices and 240 volumes covering the entire brain were acquired with repetition time (TR) = 2000 ms, echo time (TE) = 30 ms; flip angle = 90°; matrix = 256 × 256; field of view (FOV) = 24 × 24 cm^2^; and voxel size = 3.75 × 3.75 × 4 mm^3^. T1-weighted anatomical images of high resolution with 188 contiguous axial 1 mm thick slices were obtained with TR = 8.1 ms, TE = 3.7 ms; flip angle = 7°; matrix = 256 × 256; and FOV = 25.6 × 25.6 cm^2^.

### Outcomes

After the baseline investigation, alcohol-dependent patients were followed up at 1, 3 and 6 months. During the follow-up interview, all participants were asked the same question: “Have you had alcohol since your last discharge from the detoxification hospitalization?” Participants who replied “yes” were further interviewed for their first redrink time (month) and alcohol use information of the most severe month using a revised Chinese one-month version of AUDIT [31], which allows us to estimate the severity of alcohol use. The timeframe of the revised Chinese one-month version of AUDIT was modified to 1 month. According to previous studies, the optimal cutoff of AUDIT scores to classify alcohol use disorders was eight scores [29, 30]. In follow-up interviews, detoxicated alcohol-dependent patients were included in the relapse group if the AUDIT scores were ≥ 8 and the remaining alcohol-dependent patients were assigned to the nonrelapse group.

### Preprocessing of image data

Preprocessing of baseline imaging data was carried out using Data Processing Assistant for Resting-State fMRI (DPARSF) [32] software based on MATLAB (MathWorks, Natick, MA, USA). Image data with head motion parameters ≥1.5 mm or rotation ≥1.5° were excluded. Power et al. calculated head motion (framewise displacement) and found no significant difference between groups [33]. First, the first ten time points were discarded. Second, slice timing, realigning, normalization and resampling were performed in Montreal Neurological Institute (MNI) space at a resolution of 3 × 3 × 3 mm^3^. Third, we regressed out the nuisance covariates that contained Friston-24 motion parameters, cerebrospinal fluid and white matter [34]. The remaining time series were filtered through a bandpass filter within a frequency range of 0.01 to 0.1 Hz [35] and then smoothed with the Gaussian kernel (full width half maximum: 6 × 6 × 6 mm).

### Statistical analyses

#### ReHo and fALFF analyses

Two-sample *t*-test were performed on the baseline ReHo maps and fALFF between the alcohol-dependent patients and HCs as well as between the relapse group and the nonrelapse group using DPABI [36]. Age, education years and smoking status were treated as covariates. A corrected significance at the voxel level was set at *p* <  0.001 for multiple comparisons adjusted by the Family Wise Error (FWE) based on Gaussian Random Field. Clusters were defined in the Automated Anatomical Labeling (AAL) atlas [37].

#### FC analyses

We defined the brain regions showing significant differences in the ReHo or fALFF between the alcohol-dependent patients and HCs or between the relapse group and the nonrelapse group as seeds to investigate FC indicators that may be associated with alcohol dependence or relapse using two-sample *t*-test analysis. Smoking status, education years and age were corrected as covariates to control the confounding effect. Statistical inferences were the same as in the ReHo and fALFF analysis.

#### Logistic regression

To determine the neuroimaging indicators independently associated with alcohol dependence, a binary logistic regression analysis was conducted. The results showed that the FC, ReHo and fALFF values were significantly different between the alcohol dependence group and HCs in the aforementioned two-sample *t*-test analyses, where age, education years and smoking status were treated as independent variables. The logistic regression analysis to explore the rs-fMRI predictors of alcohol dependence relapse focused on the FC, ReHo and fALFF values that were significantly different between the relapse and nonrelapse groups. In addition, baseline AUDIT scores, first drinking age, years of drinking, average daily consumption in the last month, consumption at the last drinking, age, education years and smoking status were treated as potential confounding variables. The “conditional forward” model was implemented in both binary logistic regression analyses. The area under the receiver operating characteristic curve (AUC) was calculated to show the classifying or predictive power of the regression models.

The 95% confidence intervals (95% CIs), means and standard deviation (SD) were used to describe the demographic variables and outcomes. Student’s *t*-test and *χ*^*2*^ test were used to calculate the differences between groups for quantitative data and categorical data, respectively. Logistic regression analyses based on a two-tailed *α* level of 0.05 were conducted in SPSS 26.0 (IBM Corp., Armonk, NY, USA).

## Results

### Subject characteristics

The age (mean ± SD: alcohol dependence: 39.97 ± 9.00; HC: 38.03 ± 9.53) and education years (alcohol dependence: 12.76 ± 3.52; HC: 13.90 ± 3.58) were not significantly different (age: *t* = 1.22, *p* = 0.22; education years: *t* = − 1.86, *p* = 0.07) between the alcohol-dependent patients and HCs. In comparison with the HCs (38.24%), the rate of smoking among the alcohol-dependent patients (91.18%) was significantly higher (*χ2* = 41.73, *p* <  0.001). According to the baseline investigation, the mean AUDIT score of the alcohol-dependent patients was 28.13 ± 8.22. At the six-month follow-up investigation, 67 (98.53%) alcohol-dependent patients finished the follow-up research, and 35 (52.24%) of them relapsed. Between the relapse group and the nonrelapse group, differences in smoking status (relapse: 88.57%; nonrelapse: 93.75%; *χ2* = 0.10, *p* = 0.75), age (relapse: 40.97 ± 9.18; nonrelapse: 38.97 ± 8.95; *t* = 0.90, *p* = 0.37), education years (relapse: 12.51 ± 3.41; nonrelapse: 13.00 ± 3.72; *t* = − 0.56, *p* = 0.58), first drinking age (relapse: 18.71 ± 5.16; nonrelapse: 20.88 ± 5.45; *t* = − 1.67, *p* = 0.10), years of drinking (relapse: 22.49 ± 8.94; nonrelapse: 18.84 ± 8.31; *t* = 1.72, *p* = 0.09), average daily consumption in the last month (relapse: 171.94 ± 61.31; nonrelapse: 143.39 ± 79.05; *t* = 1.66, *p* = 0.10), consumption at the last drinking (relapse: 238.87 ± 678.15; nonrelapse: 151.01 ± 192.46; *t* = 0.71, *p* = 0.48) and baseline AUDIT scores (relapse: 29.02 ± 7.82; nonrelapse: 27.09 ± 8.72; *t* = 1.04, *p* = 0.3) were not statistically significant. However, the AUDIT score of the most severe month during the follow-up of the relapse group (25.26 ± 10.23) was significantly higher than that of the nonrelapse group (0.44 ± 1.34) (*t* = 14.23, *p* <  0.001). The detailed descriptive subject characteristics are presented in Table 1.

**Table 1:** Sample Demographics and their Clinical Characteristics at baseline survey and follow-up survey

	alcohol dependence (***n*** = 68)	HCs^a^ (n = 68)			Relapse^b^ (***n*** = 35)	Nonrelapse^c^ (***n*** = 32)		
	Mean ± SD^**d**^	Mean ± SD^**d**^	***t***^**e**^***/χ***^***2f***^	***P***	Mean ± SD^**d**^	Mean ± SD^**d**^	***t***^**e**^***/χ***^***2f***^	***P***
**Demographic variables**								
**Age (years)**	39.97 ± 9.00	38.03 ± 9.53	1.22	0.22	40.97 ± 9.18	38.97 ± 8.95	0.90	0.37
**Education years**	12.76 ± 3.52	13.90 ± 3.58	−1.86	0.07	12.51 ± 3.41	13.00 ± 3.72	−0.56	0.58
**Smoking status (%)**	62 (91.18%)	26 (38.24%)	41.73	< 0.001	31 (88.57%)	30 (93.75%)	0.10	0.75
**Clinical characteristics**								
**Years of drinking**	20.79 ± 8.71	–	–	–	22.49 ± 8.94	18.84 ± 8.31	1.72	0.09
**First drinking age**	19.65 ± 5.39	–	–	–	18.71 ± 5.16	20.88 ± 5.45	−1.67	0.10
**Consumption in the last month (g/day)**	158.92 ± 70.91	–	–	–	171.94 ± 61.31	143.39 ± 79.05	1.66	0.10
**Consumption at the last drinking (g)**	200.60 ± 503.35	–	–	–	238.87 ± 678.15	151.01 ± 192.46	0.71	0.48
**Score of AUDIT**^g^ **at baseline survey**	28.13 ± 8.22			–	29.20 ± 7.82	27.09 ± 8.72	1.04	0.30
**Score of AUDIT**^g^ **at follow-up survey**	–	–	–	–	25.26 ± 10.23	0.44 ± 1.34	14.23^***^	< 0.001

*HCs*^*a*^ healthy controls, *Relapse*^*b*^ relapsed among alcohol dependence and scores of AUDIT ≥8 at follow-up survey, *Nonrelapse*^*c*^ not relapsed among alcohol dependence and scores of AUDIT < 8 at follow-up survey, *SD*^*d*^ standard deviation, *t*^e^ values of student’s *t-*test, *χ*^*2f*^, values of Chi-square test, *AUDIT*^*g*^ Alcohol Use Disorder Identification Test;

### rs-fMRI indicators associated with alcohol dependence

#### ReHo

**Fig. 1:**
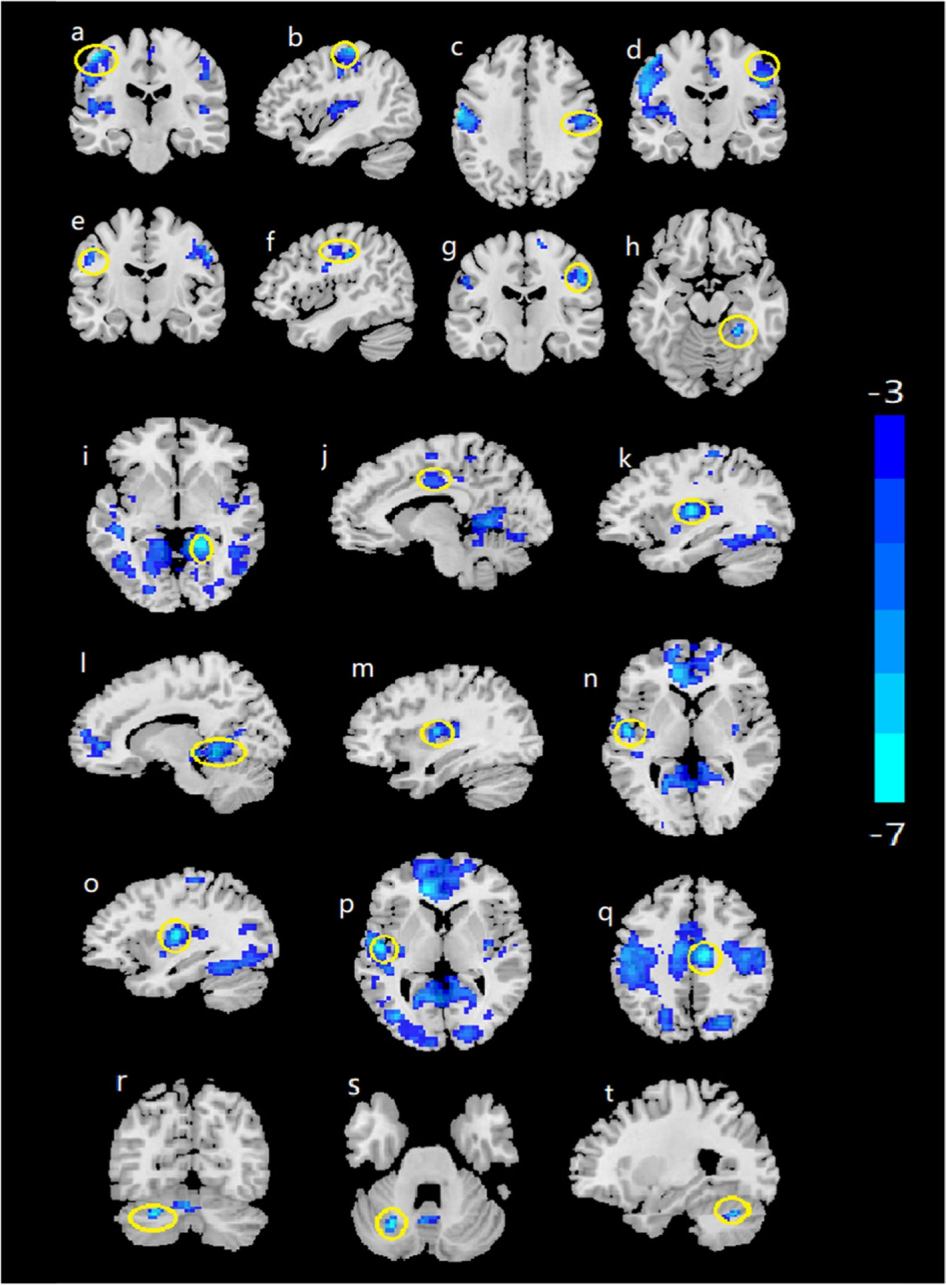
The functional indicators in the brain regions. (Alcohol dependence < HCs, relapse < nonrelapse). Compared to the HCs, the alcohol dependence group had decreased ReHo in the left postcentral (**a**), left precentral (**b**), right postcentral (**c**) and right precentral (**d**); decreased fALFF in the left postcentral (**e**), right precentral (**f**), right postcentral (**g**) and fusiform (**h**); decreased FCs of the left precentral (seed) with the right lingual (**i**), left middle cingulum (**j**) and left insula (**k**); decreased FCs of the right precentral (seed) with the right lingual (**l**), right insula (**m**) and left superior temporal (**n**); decreased FC of the left postcentral (seed) with left insula (**o**); decreased FC of the right postcentral (seed) with the left superior temporal (**p**); and decreased FC of the right fusiform (seed) with the right middle cingulum (**q**). The T map was drawn with *P* <  0.001 at the voxel level and *P*_*FWE*_ < 0.05 at the cluster level Compared to the nonrelapse group, the relapse group had: decreased FC of the left precentral (seed) with the left cerebellum (**r**, **s**, **t**). (*P* < 0.002 at the voxel level, *P*_*FWE*_ < 0.05 at the cluster level). The color bar represents the voxel T value. HCs: healthy controls. ReHo: reginal homogeneity. fALFF: fractional amplitude of low-frequency fluctuations. FCs: functional connectivities.

Compared with the HCs, the alcohol dependence group showed significant reductions in ReHo in the bilateral postcentral gyrus and the bilateral precentral gyrus (Table 2, Fig. 1).

**Table 2:** Brain regions showing significant differences in ReHo or fALFF and significantly different FCs between healthy controls and alcohol-dependent patients

	Brain regions	Hemisphere	Voxels	X	Y	Z	T
**ReHo**	**Postcentral**	**Left**	407	− 48	−21	57	− 6.538
	**Precentral**	**Left**	67	−43	−19	60	−4.424
	**Postcentral**	**Right**	149	48	−15	36	−5.247
	**Precentral**	**Right**	66	49	−14	48	−3.479
**fALFF**	**Postcentral**	**Left**	68	−51	−12	33	−4.469
	**Precentral**	**Right**	47	44	−11	46	−3.937
	**Postcentral**	**Right**	86	51	−18	36	−4.838
	**Fusiform**	**Right**	28	26	−42	−15	−4.800
**FCs of the left Precentral (seed) with**	**Lingual**	**Right**	270	18	−45	−3	−6.524
	**middle Cingulum**	**Left**	80	−9	−15	42	−3.597
	**Insula**	**Left**	75	−36	−12	12	−6.382
**FCs of the right Precentral (seed) with**	**Lingual**	**Right**	130	12	−42	−6	−5.442
	**Insula**	**Right**	48	36	−9	12	−5.310
	**superior Temporal**	**Left**	161	−51	−9	3	−5.447
**FC of the left Postcentral (seed) with**	**Insula**	**Left**	85	−36	−12	12	−7.293
**FC of the right Postcentral (seed) with**	**superior Temporal**	**Left**	286	−51	−12	3	− 5.951
**FC of the right Fusiform (seed) with**	**middle Cingulum**	**Right**	226	9	−12	39	−6.521

*ReHo* reginal homogeneity, *fALFF* fractional amplitude of low-frequency fluctuations, *FCs* functional connectivities, 𝑋, 𝑌, and 𝑍 coordinates of primary peak locations in the Montreal Neurological Institute space, A positive 𝑇 value indicates an increased ReHo, fALFF or FC, and a negative 𝑇 value indicates a decreased ReHo, fALFF or FC

#### fALFF

As shown in Table 2 and Fig. 1, alcohol-dependent subjects displayed significant reductions in the right fusiform gyrus, the bilateral postcentral gyrus and the right precentral gyrus.

#### FC

According to the ReHo and fALFF results, we defined the right fusiform, bilateral postcentral and bilateral precentral regions as seeds for the FC analyses. Compared to the HCs, some alcohol-dependent patients had significantly decreased FCs (Table 2 and Fig. 1), including the FCs of the left precentral (as seed) with the right lingual, left middle cingulum and left insula; those of the right precentral (as seed) with the right lingual, the right insula and the left superior temporal gyrus; those of the left postcentral (seed) with the left insula; those of the right postcentral (seed) with the left superior temporal gyrus; and those of the right fusiform (seed) with the right middle cingulum.

### rs-fMRI indicators independently associated with alcohol dependence

**Fig. 2:**
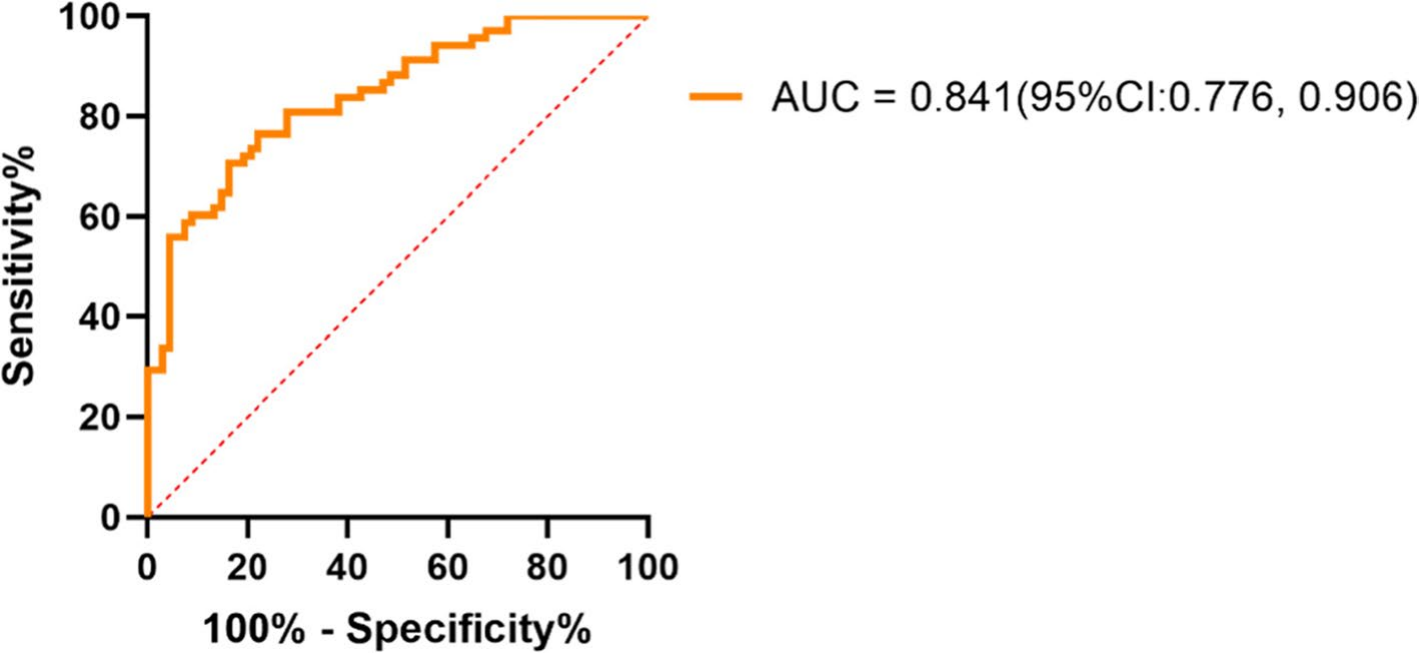
ROC curve of the logistic regression model to differentiate alcohol dependence by functional brain imaging indicators including: the ReHo of the left postcentral, the fALFF of the right fusiform, and the FC of the right fusiform (seed) with the right middle cingulum. 95%CI: 95% confidence interval; ROC: receiver operating characteristic; ReHo: reginal homogeneity; fALFF: fractional amplitude of low-frequency fluctuations; FCs: functional connectivities; AUC: area under the receiver operating characteristic curve

Three values, the ReHo of the left postcentral gyrus, the fALFF of the right fusiform gyrus and the FC between the right fusiform gyrus and the right middle cingulum, were independently associated with alcohol dependence according to the logistic regression model, which treated the significantly different FC, ReHo and fALFF values between the alcohol dependence group and HCs in the aforementioned two-sample *t*-test analyses, and age and education years as potential independent variables. When controlling smoking status as a covariate, the difference between the alcohol dependence group and HCs was also significant (Table 3). The AUC of the logistic regression models that entered the three rs-fMRI indicators was 0.841 (95% CI: 0.776, 0.906) (Fig. 2).

**Table 3:** Variables independently associated with alcohol dependence in the logistic regression models

Variables	Hemisphere	OR^**a**^ (95%CI^**b**^)	OR^**c**^ (95%CI)
**fALFF of Fusiform**	Right	0.012(0.0002, 0.675) ^***^	0.005(0.0001, 0.760) ^***^
**ReHo of Postcentral**	Left	0.024(0.004, 0.163) ^*^	0.027(0.003, 0.232) ^**^
**FC between the right Fusiform (seed) and middle Cingulum**	Right	0.022(0.001, 0.535) ***	0.02(0.0001, 0.991) ^***^
**Smoking status**	–	–	17.622(5.560, 55.853) ^*^

*OR*^*a*^ adjusted odds ratio of the multivariable binary logistic regression (forward: conditional), which set alcohol dependence (Yes vs. No) as the dependent variable, and the rest-fMRI indicators in the Table 2 (age and education years) as the independent variables, *95%CI*^*b*^ 95% confidence interval, *OR*^*c*^ adjusted odds ratio of the multivariable binary logistic regression (forward: conditional), which set alcohol dependence (Yes vs. No) as the dependent variable, and the rest-fMRI indicators in the Table 2 (age, education years and smoking status) as the independent variables, *ReHo* reginal homogeneity, *fALFF* fractional amplitude of low-frequency fluctuations, *FC* functional connectivity

*: *P* < 0.001

**: *P* < 0.01

***: *P* < 0.05

### rs-fMRI indicators associated with relapse

#### ReHo and fALFF

We did not find significant differences in ReHo or fALFF between the relapse group and the nonrelapse group.

### FC independently predicts the relapse of alcohol dependence

**Fig. 3:**
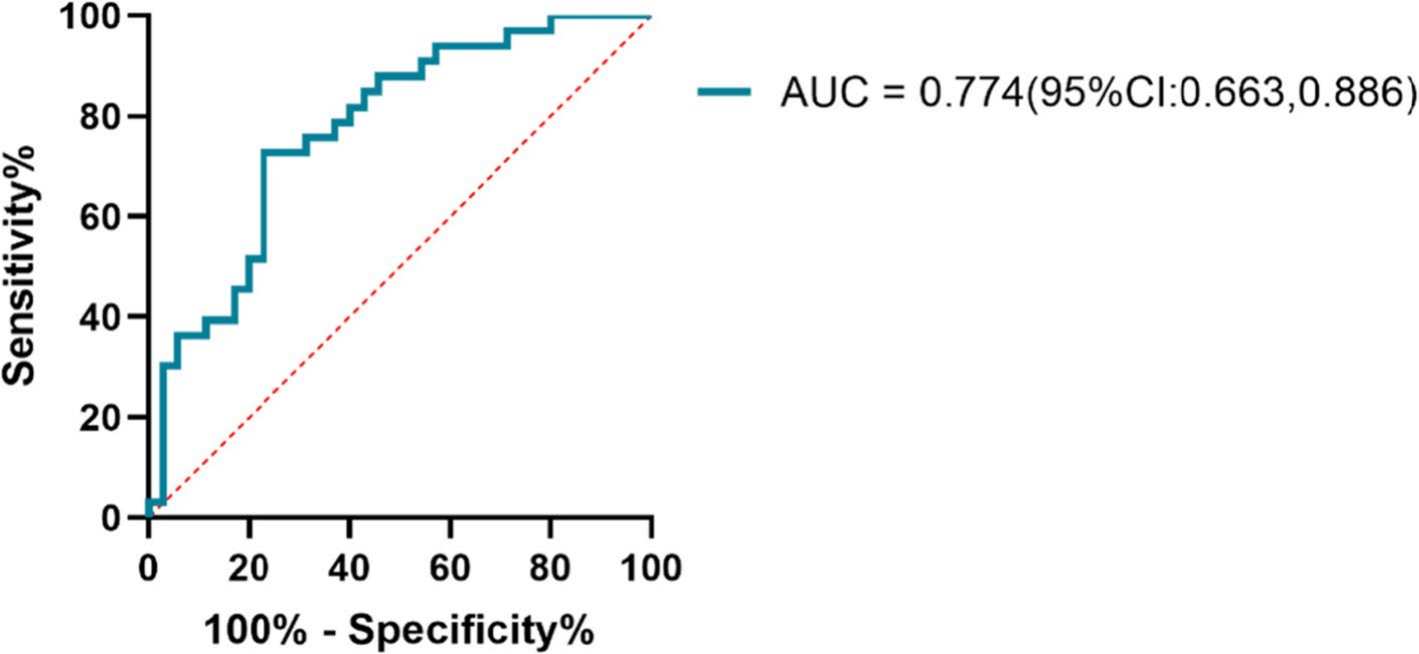
ROC curve of the logistic regression model to predict the relapse of alcohol dependence by the FC of the left precentral (seed) with the left cerebellum. 95%CI: 95% confidence interval; FC: functional connectivity; ROC: receiver operating characteristic; AUC: area under the receiver operating characteristic curve

We did not find significant differences in FCs when defining the bilateral postcentral region, the right fusiform region and the right precentral region as seeds. However, when the left precentral region was treated as a seed, a significant reduction in the baseline FC was observed between the left precentral region and the left cerebellum (*T* = − 4.445, X = − 27, Y = − 66, Z = − 30, *P*_*GRF*_ = 0.002) in the relapse group compared with the nonrelapse group (Fig. 1). Only FC was independently associated with the relapse of alcohol dependence according to the logistic regression; FC, baseline AUDIT scores, first drinking age, years of drinking, average daily consumption in the last month, consumption at the last drinking, age, education years and smoking status were treated as potential independent variables. The AUC of the regression model was 0.774 (95% CI: 0.663, 0.886) (Fig. 3).

## Discussion

We carried out data-driven research that utilized fALFF and ReHo analyses to identify the seeds of FC analyses in a study on rs-fMRI marks in early alcohol abstinence in alcohol dependence and relapse. Using this study strategy, we found that the reductions in ReHo of the left postcentral gyrus, fALFF of the right fusiform gyrus, and FC between the right fusiform gyrus and the right middle cingulum were independently associated with alcohol dependence. Furthermore, we found that the FC between the left precentral region and the left cerebellum was decreased in the relapse group, which suggested that reduction of the connection of the frontal lobe to the cerebellum might play a role in the pathogenesis of alcohol dependence relapse and might be a potential biomarker to predict relapse.

The postcentral region is associated with the primary sensory center of the brain [38, 39], while the precentral region is known as the primary motor center of the brain [40]. Both the precentral and postcentral regions are involved in the integration of information related to the sensory, motor, attention, and reward circuits [39, 41–43]. Our finding of reductions in ReHo in the left postcentral region in alcohol-dependent patients further enriched the evidence that chronic alcohol consumption might cause functional changes in the precentral and postcentral regions, which would affect sensory, motor and attention functions and brain reward mechanisms. The fusiform gyrus is a part of the temporal lobe and occipital lobe and associated with the visual cortex. A large number of previous studies have shown that the fusiform plays an important role in face recognition [44, 45]. Barriers in the process of recognition may cause the reduction of specific visual signal coding, which would decrease the probability of some identification. Therefore, our finding of decreased fALFF in the fusiform might be helpful to interpret some disorders in recognition, such as higher sensitivity to recognizing alcohol-related information, which is common among patients with severe alcohol dependence [46]. The cingulum gyrus is a structure in the limbic system that is a part of a complex network that controls emotion. In addition to regulating fatal life signs, such as heart rate and blood pressure, it plays important roles in attentional and cognitive processing [47]. In addition, the white matter of the cingulum is connected with the prefrontal cortex, and these white matter tracts may contribute to memory, emotion, and reward responses [48]. Hence, the decreased FC between the fusiform system and the cingulum in our study might be attributable to visual attention and cognitive deficiency as well as a decline in emotion control and the reward system in alcohol-dependent patients. Accordingly, the aforementioned findings, including the decreased rs-fMRI indicators of the precentral, postcentral, fusiform and cingulum regions, provided additional evidence that brain regions that play important roles in cognitive, attention, working memory, emotional control and reward systems were associated with alcohol dependence, as expected, although a data-driven (less hypothesis-oriented) investigation strategy was used in this study.

The reduction in FC of the left precentral to the left cerebellum was the only rs-fMRI indicator predictively associated with the relapse of alcohol dependence in this study, which was surprising. Previous studies have reported that decreased FC between the cerebellum and frontal lobe was associated with certain mental disorders. For example, Rodriguez et al. (2019) found hyperconnectivity between the cerebellum and precentral gyrus in patients with first-episode schizophrenia spectrum disorder, while Baxter et al. (2013) found that a group of chronic alcoholic patients showed decreased FC between the precentral gyrus and the cerebellum [49]. To our knowledge, however, the present study is the first of its kind to find that FC between the precentral gyrus and cerebellum was longitudinally associated with relapse of early abstinence alcohol dependence.

The importance of the precentral gyrus for cognition, emotion and/or reward regulation has been addressed previously [39, 42]. The cerebellum, which is known as an important CNS region associated with balance maintenance, voluntary movement coordination and motor skills learning [50], also plays important roles in cognitive, working memory and emotional regulation, as evidenced by an increasing number of recent studies [51–54]. Furthermore, the cerebellum is especially vulnerable to alcohol-related damage [55], as shown by a large number of studies in which structural and/or functional damage to the cerebellum was associated with alcohol use disorder [56, 57]. For example, several studies found that the volumes of the total cerebellum were decreased in AUD patients [58, 59]. Many studies using tasking-fMRI have demonstrated that several brain regions, including the cerebellum and primary motor areas, are significantly affected by alcohol use [60, 61]. Ritz et al. (2019) reported that patients with AUD had higher glucose absorption in cerebellar lobule VIII, which correlated with hypometabolism, especially in several frontocerebellar nodes, and cerebellar hypermetabolism was negatively related to partial hypometabolism in the frontal cortices and the premotor cortex, which might lead to working memory defects and ataxia [62]. This evidence documented by previous studies indicated that a reduction in FC between the precentral gyrus and the cerebellum associated with severe alcohol use may play important roles in cognitive, emotional and rewarding regulation associated with alcohol dependence. The finding that the reduction in FC in early alcohol abstinence alcohol-dependent patients was predictively associated with the relapse of alcohol dependence is thus scientifically explainable and may be a potential biomarker to predict alcohol dependence relapse.

The aforementioned interpretable findings notwithstanding, the present study failed to replicate that DLPFC, mPFC or NAc played significant roles in alcohol dependence, and the functions of some nuclei in the basal ganglia, such as striatal nuclei, may associated with cognition and relapse vulnerability of alcohol use disorders, as documented by previous studies [54, 63, 64]. Further research is needed to clarify whether those inconsistent findings are associated with population specific issues or with methodology differences in sample size, statistical correction for multiple tests, data-driven strategies, outcome measures and/or controlling for confounding factors.

## Limitations

First, because this study included only male participants, the results may not generalize to female alcohol-dependent patients. Some investigations have reported neuroimaging differences between females and males. Second, we did not match the smoking status between alcohol-dependent patients and HCs. Although we controlled smoking status as a covariate during the analysis process, the effect of cigarettes on brain function should not be ignored [65]. Thus, subgroups stratified by smoking status and sex should be further investigated. Third, although similar measurements of alcohol dependence relapse through interviews have been validated and used in previous studies [66, 67], future studies should further validate in this Chinese patient group with the assistance of laboratory test indicators, such as serum ethanol concentration and carbohydrate-deficient transferrin (CDT) [68]. Fourth, the confounding effect of withdrawal syndromes on brain function was not adequately controlled in the present study. Although the baseline assessment and MRI scanning were performed when the patients had experienced 5 to 12 days of hospitalized detoxification after stopping drinking and had been relieved of the withdrawal syndromes, the relief of withdrawal syndromes was based on clinicians’ judgment rather than a quantitative measurement. In addition, the present study did not adequately measure and control other possible confounding factors, such as medications for treating withdrawal syndromes, cognitive function, craving, obsessive-compulsive drinking, impulsivity, and other comorbid psychiatric symptoms such as anxiety and depression. These factors might influence brain function and/or be associated with the relapse of alcohol use disorders. Future studies, focused on not only the predictive association of brain function imaging during early abstinence of alcohol dependence with relapse but also on the neurobiological mechanism of the association, should measure those factors as adequately as possible in larger-sample investigations.

## Conclusion

This rs-fMRI study found reductions in the functional indicators of several brain regions involved in cognitive, attention, working memory, and emotion and reward regulation among male alcohol-dependent patients with early alcohol abstinence. We documented a novel finding that the decrease in FC between the precentral gyrus and the cerebellum at early alcohol abstinence was longitudinally associated with alcohol dependence relapse during follow-up and may be a potential biomarker to predict alcohol dependence relapse. These findings are meaningful for further research on the neurobiological etiology and biomarkers of relapse for alcohol dependence.

## Data Availability

The data and materials contributing to this article will be shared upon reasonable request to the corresponding author.

## Abbreviations

rs-fMRI: Resting-state functional magnetic resonance imaging
HCs: Healthy controls
ReHo: Regional homogeneity
fALFF: Fractional amplitude of low-frequency fluctuations
FCs: Functional connectivities
AUC: Area under the receiver operating characteristic curve
WHO: World Health Organization
DALYs: Disability-adjusted life years
MRI: Magnetic resonance imaging
fMRI: Functional magnetic resonance imaging
BOLD: Blood oxygenation level-dependent
DLPFC: Dorsolateral prefrontal cortex
NAc: Nucleus accumbens
mPFC: Medial prefrontal cortex
ROIs: Region of interests
DSM-IV: Diagnostic and Statistical Manual of Mental Disorders, 4th Edition
SCID-I/P: Structured Clinical Interview for the Diagnostic and Statistical Manual of Mental Disorders, 4th Edition Axis I Disorders-Patient Edition
SCID-NP: Diagnostic interview of DSM-IV Axis I Disorders-Nonpatient Edition
AUDIT: Alcohol Use Disorder Identification Test
EPI: Echo-planar imaging
TR: Repetition time
TE: Echo time
FOV: Field of view
DPARSF: Data Processing Assistant for Resting-State fMRI
MNI: Montreal Neurological Institute
FWE: Family Wise Error
AAL: Automated Anatomical Labeling
CIs: Confidence intervals
SD: Standard deviation
